# Learned Integrated Sensing Pipeline: Reconfigurable Metasurface Transceivers as Trainable Physical Layer in an Artificial Neural Network

**DOI:** 10.1002/advs.201901913

**Published:** 2019-12-06

**Authors:** Philipp del Hougne, Mohammadreza F. Imani, Aaron V. Diebold, Roarke Horstmeyer, David R. Smith

**Affiliations:** ^1^ Institut de Physique de Nice CNRS UMR 7010 Université Côte d'Azur Nice 06108 France; ^2^ Center for Metamaterials and Integrated Plasmonics Department of Electrical and Computer Engineering Duke University Durham NC 27708 USA; ^3^ Biomedical Engineering Department Duke University Durham NC 27708 USA

**Keywords:** machine learning, metasurfaces, sensing, wavefront shaping

## Abstract

The rapid proliferation of intelligent systems (e.g., fully autonomous vehicles) in today's society relies on sensors with low latency and computational effort. Yet current sensing systems ignore most available a priori knowledge, notably in the design of the hardware level, such that they fail to extract as much task‐relevant information per measurement as possible. Here, a “learned integrated sensing pipeline” (LISP), including in an end‐to‐end fashion both physical and processing layers, is shown to enable joint learning of optimal measurement strategies and a matching processing algorithm, making use of a priori knowledge on task, scene, and measurement constraints. Numerical results demonstrate accuracy improvements around 15% for object recognition tasks with limited numbers of measurements, using dynamic metasurface apertures capable of transceiving programmable microwave patterns. Moreover, it is concluded that the optimal learned microwave patterns are nonintuitive, underlining the importance of the LISP paradigm in current sensorization trends.

## Graphical abstract

Optimal wavefronts to probe a scene with microwave radiation in order to recognize objects therein are learned by integrating a model of the sensor's reconfigurable physical layer into the machine‐learning pipeline used to process the measurements. Thereby the amount of task‐relevant information obtained per measurement is maximized, enabling sensing with low latency and computational burden.

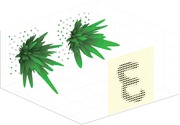

## 1 Introduction

Wave‐based sensing is of fundamental importance in countless applications, ranging from medical imaging to nondestructive testing.^1^ Currently, it is emerging as key enabling technology for “context‐aware” concepts like autonomous vehicles,^2^ ambient‐assisted living facilities^3^ and touchless human–computer interaction devices.^4^ In these context‐aware settings, an important goal is often to achieve the highest possible accuracy for a given task, such as recognizing a hand gesture, with as few measurements as possible. Minimizing the number of measurements can help improve a wide number of metrics—for example, speed, power consumption, and device complexity. It is also crucial in a variety of specific contexts—for instance, to limit radiation exposure (e.g., in X‐ray imaging), to adhere to strict timing constraints caused by radiation coherence or unknown movements in a biological context,^5^, ^6^ or to make real‐time decisions in automotive security.^2^

In all of the above applications, “active” illumination is sent out from the device to interact with the scene of interest before the reflected waves are captured by the sensor. The resulting measurements are then processed to answer a particular question. Usually, acquisition and processing are treated and optimized separately. For instance, the spatial resolution of a LIDAR system on an autonomous vehicle is often optimized to be as high as possible, while its resulting measurements are subsequently processed to detect pedestrians with as high an accuracy as possible. Recently, machine learning (ML) techniques have dramatically improved the accuracy of measurements postprocessing for complex tasks (like object recognition) without requiring explicit analytical instructions.^7^, ^8^, ^9^, ^10^, ^11^

However, to date, the physical acquisition layers of context‐aware systems have yet to reap the benefits of new ML techniques. At the same time, by separately optimizing acquisition hardware and postprocessing software, most sensing setups are not tailored to their specific sensing task. Instead, as with the LIDAR example noted above, hardware is typically optimized to obtain a high‐fidelity visual image for human consumption, thereby often ignoring available knowledge that could help to highlight information that is critical for ML‐based analysis.

Here, we address both of the above shortcomings with a new “learned sensing” paradigm for context‐aware systems that allows for joint optimization of acquisition hardware and postprocessing software. The result is a device that acquires nonimaging data that is optimized for a particular ML task. We consider the challenge of identifying settings of a reconfigurable metamaterial‐based device emitting microwave patterns that can encode as much relevant information about a scene for subsequent ML‐based classification with as few measurements as possible. However, as we will detail, this framework is general, flexible, and can impact a number of future sensing scenarios.

## 2 Illumination Strategies in Wave‐Based Sensing

The simplest approach in terms of the transceiver hardware is often to use random illuminations, for instance, by leveraging the natural mode diversity available in wave‐chaotic or multiply scattering systems.^12^, ^13^, ^14^, ^15^ Random illuminations have a finite overlap that reduces the amount of information that can be extracted per additional measurement. A truly orthogonal illumination basis, such as the Hadamard basis,^16^, ^17^, ^18^, ^19^ overcomes this (minor) issue, however, often at the cost of more complicated hardware.

These “generic” illuminations fail to efficiently highlight salient features for task‐specific sensing, which is necessary to reduce the number of required measurements. In other words, they do not discriminate between relevant and irrelevant information for the task at hand. Task‐specific optimal illumination can be challenging to determine, due to hardware constraints (e.g., few‐bit wavefront modulation), possible coupling effects between different transceivers, and in particular a lack of insight into the inner workings of the ML network (i.e., the artificial neural network, ANN) used to process the acquired data for each task.

So far, most attempts at task‐specific tailored illuminations seek to synthesize illumination wavefronts matching the expected principal components of a scene.^20^, ^21^, ^22^, ^23^ These principal component analysis (PCA) based approaches can be interpreted as a step toward optimal wave‐based sensing: they incorporate a priori knowledge about the scene but ignore a priori knowledge about measurement constraints and the task at hand (see Figure S1 in the Supporting Information). In a similar spirit, proposals to efficiently recover structured signals with learned sampling patterns have been reported.^24^

We hypothesize that wave‐based sensing can benefit from joint optimization of data acquisition and processing, making use of all available a priori knowledge. Inspired by recent works in the optical domain,^25^, ^26^, ^27^ here we interpret data acquisition as a trainable physical layer that we integrate directly into an ANN pipeline. By training the ANN with a standard supervised learning procedure, we can simultaneously determine optimal illumination settings to encode relevant scene information, along with a matched postprocessing algorithm to extract this information from each measurement—automatically taking into account any constraints on transceiver tuning, coupling and the number of allowed measurements.

## 3 LISP for Object Recognition with Dynamic Metasurface Apertures

As noted above, we apply our concept to classification tasks with microwave sensors which are a crucial stepping stone toward numerous context‐aware systems. Microwave sensors can operate through optically opaque materials such as clothing, are not impacted by external visible lighting and scene color, minimally infringe upon privacy (unlike visual cameras) and may eventually help sense through fog, smoke, and “around‐the‐corner.”^4^

We focus on optimally configuring dynamic metasurface hardware, a promising alternative to more traditional antenna arrays for beam‐forming and wavefront shaping.^16^ Dynamic metasurfaces are electrically large structures patterned with metamaterial elements that couple the modes of an attached waveguide or cavity to the far field.^28^, ^29^ Reconfigurability is achieved by individually shifting each metamaterial element's resonance frequency, for instance, with a PIN diode.^30^ Compared to a traditional antenna array that uses amplifiers and phase shifters, the inherent analog multiplexing makes dynamic metasurface hardware much simpler, less costly and easier to integrate in many applications (see Section IV in the Supporting Information).

**Figure 1:**
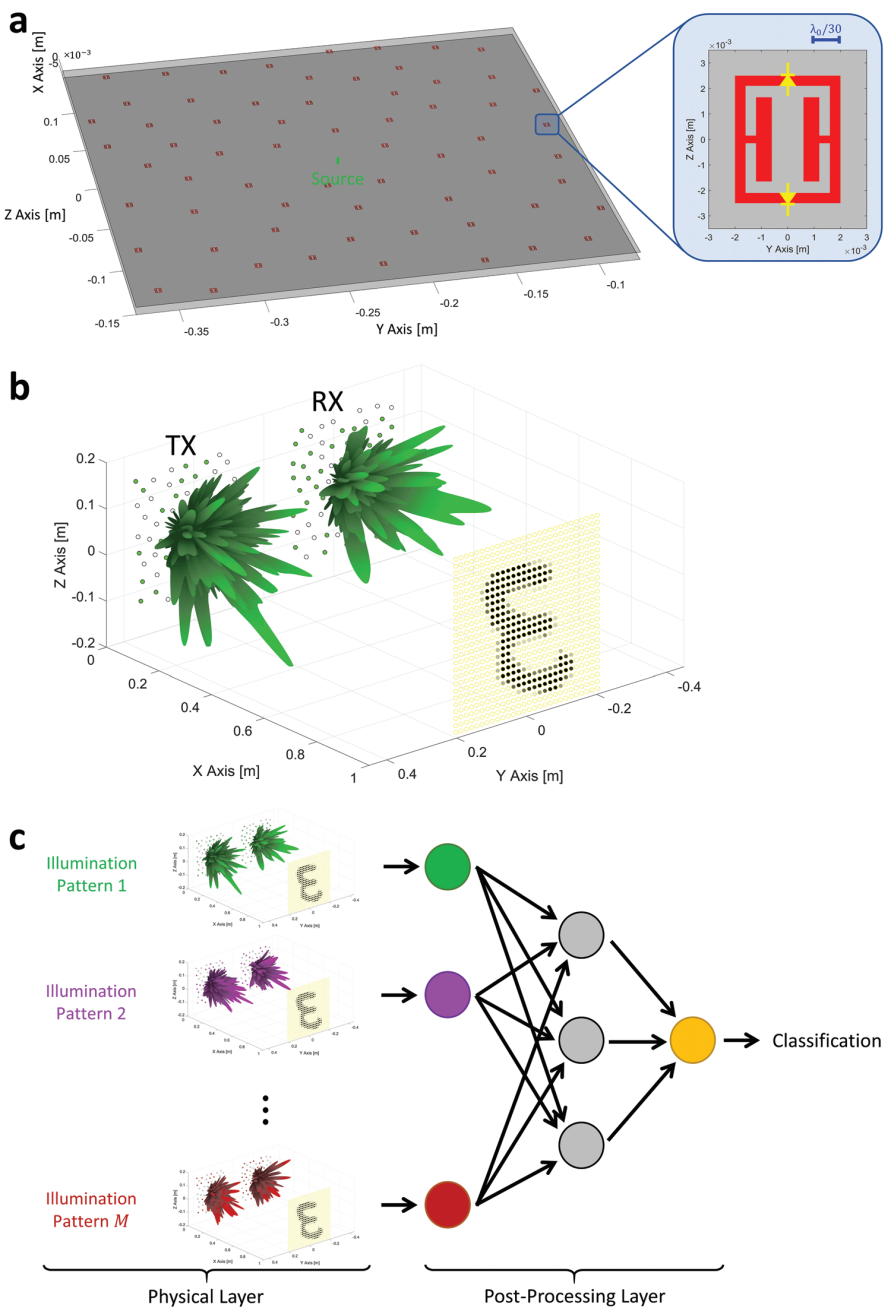
Schematic overview of operation principle. a) Dynamic metasurface. *N* = 64 tunable metamaterial elements are patterned into the upper surface of a planar waveguide. The inset shows the geometry of an example cELC metamaterial element that could be used in combination with PIN diodes (location and orientation indicated in yellow) to reconfigure the element. The waveguide is excited by the indicated line source. b) Sensing setup. The scene consists of a metallic digit in free space that is illuminated by a TX metasurface and the reflected waves are captured by a second RX metasurface. c) Sensing protocol. The scene is illuminated with *M* distinct TX‐RX metasurface configurations, yielding a 1 × *M* complex‐valued measurement vector that is processed by an artificial neural network consisting of fully connected layers. The output is a classification of the scene.

The reconfigurable metasurface aperture that we consider for the generation of shaped wavefronts is depicted in **Figure** **1**a. It consists of a planar metallic waveguide that is excited by a line source (a coaxial connector in a practical implementation). *N* metamaterial elements are patterned into one of the waveguide surfaces to couple the energy to free space. An example of a metamaterial element that could be used is the tunable complimentary electric‐LC (cELC) element^30^ shown in the inset of Figure 1a. A possible tuning mechanism to individually configure each metamaterial element's radiation properties involves diodes. Then, the cELC element is resonant or not (equivalently, radiating or not radiating) at the working frequency of *f*_0_ =  10 GHz depending on the bias voltage of two PIN diodes connected across its capacitive gaps. The *N* metamaterial elements are randomly distributed within a chosen aperture size (30 cm  ×  30 cm) but a minimum distance between elements of one free‐space wavelength is imposed.

## 4 Operation Principle

To demonstrate our proposed learned integrated sensing pipeline (LISP), we jointly optimize the illumination and detection properties of dynamic metasurface transceivers, along with a simple neural network classifier, for the task of scene classification. We consider the dummy task of recognizing “handwritten” metallic digits in simulation. Replacing this dummy task with a more realistic scenario, such as concealed‐threat detection, hand‐gesture recognition, or fall detection, is conceptually straightforward. To construct the LISP, we first formulate an analytical forward model of the measurement procedure. Second, we allow certain key parameters within the “physical” forward model to act as unknown weights (here the reconfigurable resonance frequency of each metamaterial element) that we aim to optimize over. Third, we merge this weighted physical model into an ANN classifier, and use supervised learning to jointly train the unknown weights in both to maximize the system's classification accuracy.

**Figure 2:**
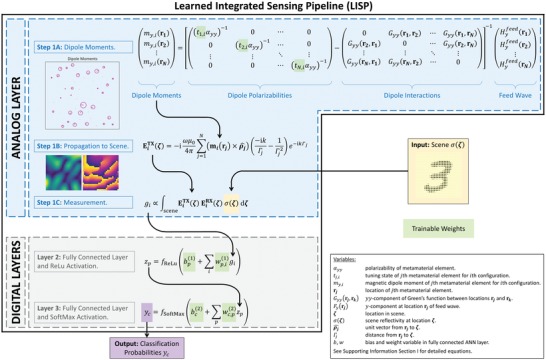
Learned integrated sensing pipeline (LISP). The analog (physical) layer corresponds to the sensing setup introduced in Figure 1b. A scene is illuminated with a dynamic metasurface, and the reflected waves are captured with a second metasurface. The analytical forward model for the analog layer consists of three steps. First, each metamaterial element's magnetic dipole moment is calculated for a given metasurface configuration. The inset shows an example of calculated dipole moments which are represented as phasors, with the radius of the circle being proportional to their amplitude, and the line segment showing their phase. The circles are centered on the physical location of each metamaterial element. Second, the field radiated by these dipoles to the scene is computed. The inset shows amplitude (left) and phase (right) of a sample field illuminating the scene. Third, the measurement is evaluated. Note that the figure contains the equations for Steps 1A and 1B only for the TX metasurface, for the sake of clarity; the RX equations are analogous. The measurement vector, consisting of complex‐valued entries corresponding to different configurations of the TX‐RX metasurfaces, is then processed by two fully connected layers consisting of 256 and 10 neurons, respectively. Finally, a classification of the scene is obtained as output. Trainable weights in our hybrid analog–digital ANN pipeline are both in the analog and the digital layers and highlighted in green. During training, these are jointly optimized via error back propagation.

We establish an analytical forward model of our sensor's physical layer built upon a compact description of the metamaterial elements as dipoles. This dipole approximation is facilitated by the intrinsic sub‐wavelength nature of the metamaterial elements. As detailed in Section I in the Supporting Information, our forward model consists of three steps: i) extracting each metamaterial element's dipole moment while taking tuning state and interelement coupling into account;^31^ ii) propagating the radiated field to the scene;^32^ iii) evaluating the scattered field. **Figure** **2** summarizes the key equations corresponding to these three steps. The ability to predict the patterns radiated by metasurface transceivers with a coupled‐dipole model has previously been verified in full‐wave simulations^31^ and experiments.^33^

A single measurement, depending on various factors such as the sensing task's complexity, the type of scene illumination but also the signal‐to‐noise ratio, may not carry enough information to successfully complete the desired sensing task.^34^ Hence, in general, we illuminate the scene with *M* distinct patterns. Each pattern corresponds to a specific pair of TX and RX metasurface configurations. Since our scheme is monochromatic, each measurement yields a single complex value *g_i_*. As shown in Figure 1c, our 1 × *M* complex‐valued measurement vector is fed into a processing ANN. The latter consists of two fully connected layers, as shown in Figure 2 (see Section II in the Supporting Information for details).

We can now assemble our pipeline consisting of an analog and two digital layers as outlined above (Figure 2). The input, a scene, is injected into the analog layer which contains trainable weights and is moreover highly compressive. The output from the analog layer, the measurement vector, continues to be processed by the digital layers which contain trainable weights as well. The final digital layer's output is the classification of the scene. By jointly training the analog and the digital weights, we identify illumination settings that optimally match the constraints and processing layer. Importantly, we expect the ANN to find a heuristically optimal compromise also in cases where the aperture size is small and few tunable elements are available, meaning that PCA modes cannot be synthesized accurately, and when the number of measurements is very limited, meaning that not all significant PCA modes can be probed.

The weights are trained by error backpropagation^35^ with 60 000 sample scenes from the MNIST dataset. Using a temperature parameter,^25^ we ensure that the learned physical weights (*t*_*j*,*i*_ in Figure 2) are binary, as required by the considered metasurface hardware. In order to compare our LISP with the benchmarks of orthogonal and PCA‐based illuminations, we solve the corresponding inverse design problems by only taking the analog layer of our pipeline and defining a cost function based on the scene illuminations (rather than the classification accuracy). Details are provided in Section II in the Supporting Information.

## 5 Sensing Performance

**Figure 3:**
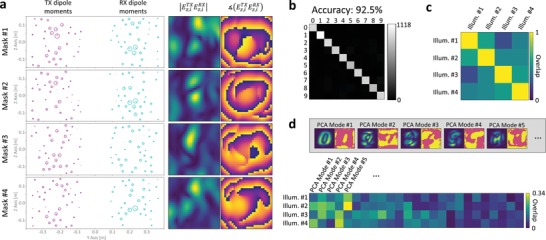
Analysis of LISP illumination patterns for a single realization with *M* = 4. a) For each of the four masks, the corresponding TX and RX dipole moments, and the magnitude and phase of the corresponding scene illuminations $E_{z,i}^{\mathrm{TX}}E_{z,i}^{\mathrm{RX}}$, *i*∈{1,2,3,4}, are shown. The dipole moment representations are as in the inset of Figure 2. The magnitude maps are normalized individually, the phase maps have a color scale from −π to π. b) Confusion matrix evaluated on an unseen test dataset of 10 000 samples. This realization achieved 92.5% classification accuracy. c) Mutual overlap of the four scene illuminations. The average over the off‐diagonal entries of the overlap matrix is 0.45. d) Overlap of the four scene illuminations with the first 25 PCA modes. Note that the color scale's maximum is 0.34 here (i.e., well below unity). The inset shows magnitude and phase of the first five PCA modes.

We begin by considering a single realization with *M* = 4 measurements and *N* = 64 metamaterial elements per metasurface. The dipole moments and scene illuminations corresponding to the four learned metasurface configurations are displayed in **Figure** **3**a. The performance metric to evaluate the sensing ability is the average classification accuracy on a set of 10 000 unseen samples. The confusion matrix in Figure 3b specifically shows how often a given digit is classified by the ANN as one of the ten options. The strong diagonal (corresponding to correctly identified digits) reflects the achieved average accuracy of 92.5%. The off‐diagonal entries of the confusion matrix are uniformly weak, so the ANN does not get particularly confused by any two classes.

**Figure 4:**
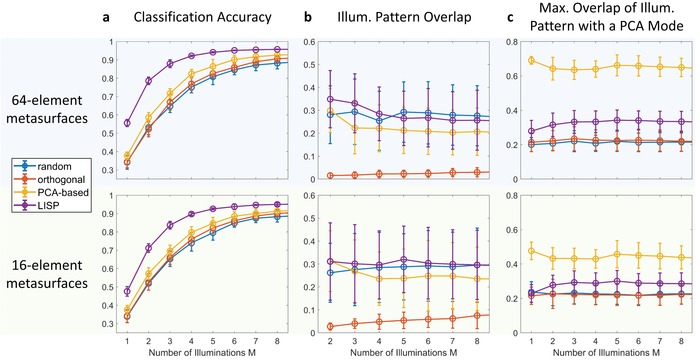
Systematic analysis of LISP performance and illumination patterns. a) Comparison of the sensing performance with the LISP illumination settings with three benchmarks: random, orthogonal and PCA‐based scene illuminations. b) Analysis of the mutual overlap between distinct patterns in a series of scene illuminations. c) Analysis of the maximum overlap of the scene illuminations with a PCA mode. All data points are averaged over 40 realizations. Error bars indicate the standard deviation.

In **Figure** **4**a, we study the sensing performance more systematically for different values of *M* and *N*. Since the ANN weights are initialized randomly before training, we conduct 40 realizations for each considered parameter combination. Averaging over realizations allows us to focus on the role of *M* and *N* without being sensitive to a given realization. Moreover, we can see the extent to which different realizations converge to similar results.

Random illuminations yield the worst performance out of the four considered paradigms. Orthogonal illuminations yield a marginal improvement over random illuminations as the number of measurements *M* gets larger, since the nonoverlapping illuminations can extract marginally more information about the scene. Yet the information that random and orthogonal illuminations encode in the measurements is not necessarily task‐relevant. The PCA‐based approach presents a noticeable performance improvement over generic illuminations because it makes use of a priori knowledge about the scene; yet it remains well below the attainable performance with our LISP because it ignores available a priori knowledge about the measurement constraints and the task. The PCA‐based approach is quite sensitive to the number of tunable elements in the dynamic metasurface (keeping the same aperture size), since beam synthesis works better with more degrees of freedom. In contrast, using only 16 instead of 64 metamaterial elements yields almost identical sensing performance with our LISP, highlighting our LISP's ability to successfully perform sensing tasks with a very light hardware layer.

For *M* ≤ 5, our scheme yields gains in accuracy of the order of 10–15% which is a substantial improvement in the context of classification tasks.^7^ The performance using our learned illumination settings saturates around *M* = 5 at 95%, meaning that we manage to extract all task‐relevant information to distinguish ten classes with only five measurements. The compression is enabled by the sparsity of task‐relevant features in the scene. Yet our scene is not sparse in the canonical basis: our region of interest corresponds to the size of the metallic digits. Unlike traditional computational imaging schemes that multiplex all scene information across measurements taken with a set of diverse (random, orthogonal) illumination patterns, the compression here comes from the discrimination between relevant and irrelevant information in a dense scene. Our LISP thus achieves a significant dimensionality reduction by optimally multiplexing task‐relevant information from different parts of the scene in the analog layer. The dimensionality reduction brings about a double advantage with respect to timing constraints: taking fewer measurements takes less time, and moreover less data has to be processed by the digital layers. In our (not heavily optimized) ANN architecture, the computational burden of the first digital ANN layer is directly proportional to the number of measurements *M*.

A striking difference in the performance fluctuations, evidenced by the error bars in Figure 4a, is also visible. While the performance of our LISP does not present any appreciable fluctuations for *M* ≥ 4, all other benchmark illumination schemes continue to fluctuate by several percent of classification accuracy. Our scheme's performance is thus reliable whereas any of the other benchmarks in any given realization may (taking the worst‐case scenario) yield a classification accuracy several percent below its average performance. Performance reliability of a sensor is important for deployment in real‐life decision‐making processes.

## 6 Analysis of Learned Scene Illuminations

The inferior performance of orthogonal and PCA‐based illuminations suggests that the task‐specific learned LISP illuminations do not trivially correspond neither to a set of optimally diverse illuminations nor to the principal components of the scene. To substantiate this observation, we analyze the scene illuminations in more detail. First, within a given series of *M* illuminations, we compute the mutual overlap between different illuminations. In the following, we define the overlap *O* of two scene illuminations *A*(***ζ***) and *B*(***ζ***) as

(1) $$O\;\left(A,B\right)=\left|\frac{\int_{\mathrm{scene}}A^{†}Bd\zeta }{\sqrt{\int_{\mathrm{scene}}A^{†}A\;d\zeta \int_{\mathrm{scene}}B^{†}B\;d\zeta }}\right|$$

where † denotes the conjugate transpose operation. An example overlap matrix for *M* = 4 is shown in Figure 3c.

In Figure 4b, we present the average illumination pattern overlap for all four considered paradigms. For the case of orthogonal illuminations, the overlap is very close to zero, indicating that our inverse metasurface configuration design worked well. The inverse design works considerably better with *N* = 64 as opposed to *N* = 16, and of course the more illuminations we want to be mutually orthogonal the harder the task becomes. While radiating orthogonal wavefronts with the dynamic metasurface is not the best choice for sensing, it may well find applications in wireless communication.^36^ For the case of random illuminations, the mutual overlap is constant at 28.5% and independent of *N*. This is indeed the average overlap of two random complex vectors of length 10, 10 corresponding roughly to the number of independent speckle grains in the scene—see Figure 3a.

The mutual overlap of PCA‐based illuminations, except for very low *M*, saturates around 21.5% and 24.5% for *N* = 64 and *N* = 16, respectively, and is hence lower than that of random illuminations. In principle, if beam synthesis worked perfectly, the PCA‐based patterns should not overlap at all since PCA modes are orthogonal by definition. The more degrees of freedom *N* we have, the better the beam synthesis works, and consequently the lower the mutual overlap of PCA modes is. For the LISP scene illuminations, the average mutual overlap is comparable to that of random scene illuminations. We hence conclude that the diversity of the scene illuminations is not a key factor for the extraction of task‐relevant information.

Next, we investigate to what extent the scene illuminations overlap with PCA modes. An example of the overlap with the first 25 PCA modes is provided in Figure 3d. In Figure 4c, we present the average of the maximum overlap that a given illumination pattern has with any of the PCA modes. For random and orthogonal illuminations, irrespective of *N* and *M*, this overlap is around 20% and thus insignificant, as expected. For PCA‐based illuminations we have performed beam synthesis to precisely maximize this overlap. We achieve (65.0 ± 1.9)% with *N* = 64 and (44.1 ± 1.4)% with *N* = 16. The ability to synthesize the PCA modes is thus very dependent on the number of metamaterial elements, and these results demonstrate that the PCA‐based approach is suitable only for scenarios where *N* is large. This observation is a further argument for the attractiveness of our approach in applications with very limited aperture size and tunability like automotive RADAR. In fact, since the PCA‐based approach also requires an analytical forward model, one may as well choose the superior performance of our LISP proposal in any scenario where the PCA‐based approach could be employed. Moreover, training with our approach is faster since all weights are optimized simultaneously, as opposed to first solving *M* inverse design problems and then training the digital weights.

The overlap of the LISP illuminations with PCA modes is around 30% and thus notably larger than for random or orthogonal illuminations but also notably lower than what can be achieved if one seeks PCA modes. Interestingly, the maximum overlap with a PCA mode is lower for *M* = 1 and *M* = 2. We conclude that the optimal illumination patterns identified by our LISP cannot simply be explained as corresponding to PCA modes, or to be a good approximation thereof, notably for small *M*. PCA modes do not account for physical layer constraints, nor the task‐specific inner workings of the nonlinear digital layers.

## 7 Conclusions

We have demonstrated that integrating the physical layer of a reconfigurable wave‐based sensor into its artificial‐neural‐network (ANN) pipeline substantially enhances the ability to efficiently obtain task‐relevant information about the scene. The jointly optimized analog and digital layers optimally encode relevant information in the measurements, converting data acquisition simultaneously into a first layer of data processing. We considered classification tasks with dynamic metasurface transceivers and observed 10–15% accuracy gains with limited numbers of measurements compared to current paradigms, as well as improved reliability. A thorough analysis of the learned illumination patterns revealed that they cannot be anticipated from outside the ANN pipeline. Further research should now focus on experimental demonstrations with real‐life tasks, and could also consider ways to enhance the robustness to fluctuations in calibration parameters^8^ as well as to make on‐the‐fly use of knowledge from previous measurements^37^ (see Section V in the Supporting Information).

## Conflict of Interest

The authors declare no conflict of interest.

## Author Contributions

The project was initiated and conceptualized by P.d.H. with input from M.F.I. and R.H. The project was conducted and written up by P.d.H. All authors contributed with thorough discussions.

## Supporting information

Supporting Information
