# Supplementary material for: Learned Integrated Sensing Pipeline: Reconfigurable Metasurface Transceivers as Trainable Physical Layer in an Artificial Neural Network
Source: Adv Sci (Weinh). 2019 Dec 6;7(3):1901913. doi: 10.1002/advs.201901913 (PMC7001623; doi:10.1002/advs.201901913)
Supplement: Supplementary file 1 — Supporting Information [file ADVS-7-1901913-s001.pdf]

Copyright WILEY-VCH Verlag GmbH & Co. KGaA, 69469 Weinheim, Germany, 2019.

Supporting Information

for *Adv. Sci.,* DOI: 10.1002/advs.201901913

Learned Integrated Sensing Pipeline: Reconfigurable Metasurface Transceivers as Trainable Physical Layer in an Artificial Neural Network

Philipp del Hougne, Mohammadreza F. Imani, Aaron V. Diebold, Roarke Horstmeyer, and David R. Smith*

Supporting Information

Learned Integrated Sensing Pipeline: Reconfigurable Metasurface Transceivers as Trainable Physical Layer in an Artificial Neural Network

Philipp del Hougne, Mohammadreza F. Imani, Aaron V. Diebold, Roarke Horstmeyer, David R. Smith*

I. Analytical Forward Model of the Physical Layer

The intrinsic sub-wavelength nature of the radiating metamaterial elements enables a compact description in terms of coupled dipoles[1–4]. Ultimately, we will directly link a given metasurface configuration (specifying which elements are radiating) to the radiated field. Note that the possibility to analytically do so is a key advantage of this metasurface hardware over alternative devices with reconfigurable radiation pattern such as leaky reconfigurable wave-chaotic three-dimensional cavities[5–7]. For the latter, one could learn forward models based on near-field scans of radiated fields[8,9]; yet the number of required equivalent source dipoles[10] (even with a coarse half-wavelength sampling of the aperture) is much higher than in our case where it is simply *N*. The ability of the coupled-dipole model that we outline in the following to predicted the wave patterns radiated by metasurface transceivers has been verified through full-wave simulations[11] and more recently in a simplified setup (slots instead of cELC metamaterial elements) also experimentally[12].

Dipole Moments. – Generally speaking, a point-like scatterer's magnetic dipole moment $\mathbf{m}$ is related to the incident magnetic field $\mathbf{H^{loc}}$ via the scatterer's polarizability tensor $\bar{\bar{\alpha}}$:

$$\mathbf{m}(\mathbf{r}) = \bar{\bar{\alpha}}\, \mathbf{H^{loc}}(\mathbf{r})\,, \qquad \text{(S1)}$$

where $\mathbf{r}$ denotes the scatterer's location. In our case, using surface equivalent principles, an effective polarizability tensor of the metamaterial element embedded in the waveguide structure can be extracted[13]. For the metamaterial element that we consider, only the α_{yy} component is significant. In the following, we use $\alpha_{yy} = (1.5 - 3.5i) \times 10^{-7}\mathrm{m}^3$ which is a typical polarizability value for a tunable cELC element[13]. The tuning state of a given metamaterial element can be encoded in its polarizability: if the element is "off", its polarizability (at the working frequency) is zero and hence it does not radiate any energy. We thus use $t_{j,i}\alpha_{yy}$ as effective polarizability, where $t_{j,i} \in \{0,1\}$ is the tuning state of the *j*th metamaterial element in the *i*th metasurface configuration.

The local magnetic field $\mathbf{H^{loc}}$ that excites the metamaterial element is a superposition of the feed wave and the fields scattered off the other metamaterial elements. $\mathbf{H^{loc}}$ in Equation S1 is thus itself a function of the metamaterial elements' magnetic dipole moments, yielding the following system of coupled equations[11]:

$$H_y^{loc}(\mathbf{r_j}) = \left(t_{j,i}\alpha_{yy}\right)^{-1} m_y(\mathbf{r_j}) \qquad \text{(S2a)}$$

$$H_y^{loc}(\mathbf{r_j}) = H_y^{feed}(\mathbf{r_j}) + \sum_{j\neq k} G_{yy}(\mathbf{r_j},\mathbf{r_k})\, m_y(\mathbf{r_k})\,, \qquad \text{(S2b)}$$

where Equation S2a is a rearranged version of Equation S1 and the index i identifies the ith metasurface configuration. Equation 2b expresses the local field at location $\mathbf{r_j}$ as sum of the feed wave H_y^{feed} at $\mathbf{r_j}$ and the individual contributions from each of the other metamaterial elements at $\mathbf{r_k} \neq \mathbf{r_j}$ via the Green's functions $G_{yy}(\mathbf{r_j}, \mathbf{r_k})$. Explicit expressions for $H_y^{feed}(\mathbf{r_j})$ and $G_{yy}(\mathbf{r_j}, \mathbf{r_k})$ are provided at the end of this section. To solve Equation S2 for the magnetic dipole moments we rewrite it in matrix form and perform a matrix inversion, as shown in Step 1A in the top line of Figure 2:

$$\mathbf{M_i} = \{\mathbf{A_i} - \mathbf{G}\}^{-1}\mathbf{F}\,, \qquad \text{(S3)}$$

where $\mathbf{M_i} = [m_{y,i}(\mathbf{r_1}), m_{y,i}(\mathbf{r_2}), \dots, m_{y,i}(\mathbf{r_N})]$, $\mathbf{A_i} = \mathrm{diag}\left(\left[(t_{1,i}\alpha_{yy})^{-1}, (t_{2,i}\alpha_{yy})^{-1}, \dots, (t_{N,i}\alpha_{yy})^{-1}\right]\right)$ and $F = [H_y^{feed}(\mathbf{r_1}), H_y^{feed}(\mathbf{r_2}), \dots, H_y^{feed}(\mathbf{r_N})]$. The off-diagonal entry (j,k) of $\mathbf{G}$ is $G_{yy}(\mathbf{r_j}, \mathbf{r_k})$, and the diagonal of $\mathbf{G}$ is zero since the self-interaction terms are incorporated into the effective polarizabilities in $\mathbf{A_i}$.

Due to the metamaterial element interactions via the $G_{yy}(\mathbf{r_j}, \mathbf{r_k})$ term, the mapping from tuning state to dipole moments is not linear. This is visualized with an example in Figure S2. Thus, ultimately the mapping from tuning state to radiated field cannot be linear, which is a substantial complication for most beam synthesis approaches; in our LISP this does not pose any additional complication.

Propagation to Scene. – We now identify the wavefront impacting the scene for a given metasurface configuration. The sensing setup we consider is depicted in Figure 1b. A transmit (TX) metasurface illuminates the scene. The scene, in our case, consists of a planar metallic digit of size 40 cm × 40 cm in free space that is to be recognized at a distance of 1 m.

To compute the ith incident TX wavefront (corresponding to the ith metasurface configuration) at a location $\boldsymbol{\zeta}$ in the scene, we superimpose the fields radiated by each of the N metamaterial element dipoles[10,14]:

$$\mathbf{E_i^{TX}}(\boldsymbol{\zeta}) = \frac{-i\omega\mu_0}{4\pi}\sum_{j=1}^{N}\left(\mathbf{m_i}(\mathbf{r_j}) \times \hat{\boldsymbol{\rho}}_{\mathbf{j}}\left(\frac{-ik}{\Gamma_j} - \frac{1}{\Gamma_j^2}\right)e^{-ik\Gamma_j}\right), \qquad \text{(S4)}$$

where $\omega = 2\pi f_0$, $\Gamma_j = |\boldsymbol{\zeta} - \mathbf{r_j}|$, $\hat{\boldsymbol{\rho}}_{\mathbf{j}}$ is a unit vector parallel to $\boldsymbol{\zeta} - \mathbf{r_j}$ and $\mathbf{m_i}(\mathbf{r_j})$ is the magnetic dipole moment of the jth metamaterial element in the ith metasurface configuration. Equation 4 is the second step of our analytical forward model, as shown in Step 1B in Figure 2. Since the magnetic dipole moments only have a significant y-component, the radiated electric field's dominant component is along z.

Measurement. – We now identify the portion of TX fields that is reflected off the scene and collected by the second receiving (RX) metasurface, as shown in Figure 1b. Since our scene is flat, parallel to the plane containing the TX and RX metasurface transceivers and reflections are primarily specular, the first Born approximation is a suitable description: the field reflected at location $\boldsymbol{\zeta}$ in the scene is $\mathbf{E}_\mathbf{i}^{\mathbf{TX}}(\boldsymbol{\zeta})\,\sigma(\boldsymbol{\zeta})$, where σ is the scene reflectivity. The RX metasurface captures specific wave forms depending on its configuration; their shape is essentially defined via a time-reversed version of Equation S4. The complex-valued signal measured for the *i*th pair of TX-RX configurations is thus[10]

$$g_i \propto \int_{scene} \mathbf{E}_\mathbf{i}^{\mathbf{TX}}(\boldsymbol{\zeta}) \cdot \mathbf{E}_\mathbf{i}^{\mathbf{RX}}(\boldsymbol{\zeta})\, \sigma(\boldsymbol{\zeta})\, \mathrm{d}(\boldsymbol{\zeta}) \,. \qquad (S5)$$

This is the third and final step of our analytical forward model, shown as Step 1C in Figure 2. Note that the scene $\sigma(\boldsymbol{\zeta})$ is ultimately sampled by $\mathbf{I_i}(\boldsymbol{\zeta}) = \mathbf{E}_\mathbf{i}^{\mathbf{TX}}(\boldsymbol{\zeta}) \cdot \mathbf{E}_\mathbf{i}^{\mathbf{RX}}(\boldsymbol{\zeta})$; when loosely referring to "scene illumination" in this work, we mean this product of $\mathbf{E}_\mathbf{i}^{\mathbf{TX}}(\boldsymbol{\zeta})$ and $\mathbf{E}_\mathbf{i}^{\mathbf{RX}}(\boldsymbol{\zeta})$. In practice, to compute the integral, we discretize the 28×28 reflectivity map of the scene (the separation of adjacent pixel centers is half a wavelength, there is no gap between the edges of neighboring pixels) with one point per pixel, as seen in Figure 1b. Each point's reflectivity value $\sigma(\boldsymbol{\zeta})$ is a gray-scale real value determined by the corresponding handwritten digit's reflectivity map.

The analytical expression for $H_y^{feed}(\mathbf{r})$ reads[11]

$$H_y^{feed}(\mathbf{r}) = \frac{iI_e k}{4} \mathrm{H}_1^{(2)}(k|\mathbf{r}-\mathbf{r_0}|)\sin(\theta), \qquad (S6)$$

where i is the imaginary unit, I_e is the amplitude of the electric line source generating the feed wave (taken to be 1 A in our work), $k = 2\pi f_0/c$ is the propagation constant of the fundamental mode inside the waveguide, $\mathrm{H}_1^{(2)}$ is a first order Hankel function of the second kind, $\mathbf{r_0}$ is the position of the source, and θ is the circumferential angle around the source measured from the y-axis. The space inside the waveguide we consider is empty (not filled with a substrate).

The inter-element coupling $G_{yy}(\mathbf{r_i}, \mathbf{r_j})$ consist of two components – interactions via the waveguide (WG) and interactions via free space (FS):

$$G_{yy}(\mathbf{r_i}, \mathbf{r_j}) = G_{yy}^{WG}(\mathbf{r_i}, \mathbf{r_j}) + G_{yy}^{FS}(\mathbf{r_i}, \mathbf{r_j}). \qquad (S7)$$

The waveguide interaction component reads[11]

$$G_{yy}^{WG}(\mathbf{r_i}, \mathbf{r_j}) = -\frac{ik^2}{8h}\left(\mathrm{H}_0^{(2)}(kR) - \cos(2\phi)\,\mathrm{H}_2^{(2)}(kR)\right), \qquad (S8)$$

where $R = |\mathbf{r_i} - \mathbf{r_j}|$ and ϕ is the angle of the vector from $\mathbf{r_i}$ to $\mathbf{r_j}$, and h is the height of the waveguide. We use $h = 5.21$ mm in our simulations. The free space interaction is given by[14,15]

$$G_{yy}^{FS}(\mathbf{r_i}, \mathbf{r_j}) = g(R)\left(k^2 + \frac{ik}{R} - \frac{1 + k^2\Delta^2}{R^2} - \frac{3ik\Delta_y^2}{R^3} + \frac{3\Delta_y^2}{R^4}\right), \qquad (S9)$$

where $\Delta_y = y_i - y_j$ and $g(R) = 2\frac{e^{-ikR}}{4\pi R}$. The factor 2 in the expression for $g(R)$ originates from self-images of the metamaterial elements due to the waveguide's upper metallic plate.

II. Further Details of Artificial Neural Network Algorithm and Parameters

In this section, for the sake of reproducibility, we detail the implementation of our method using a low-level API of the open source machine learning library TensorFlow[16]. The ANN architecture has been described in the main text. The measurements are fed into a first fully connected layer consisting of 256 neurons with ReLu activation. This is followed by a second fully connected layer made up of 10 neurons with SoftMax activation, yielding a normalized probability distribution as output. The highest value therein corresponds to the classification result (one digit between "0" and "9"). This architecture was chosen without much optimization and still performs quite well; its performance was observed to not significantly depend on the chosen hyperparameters, such as the number of neurons. We use the float32 and complex64 data types for real and complex valued variables, respectively.

Since the metamaterial elements are separated by at least a wavelength, the arguments of the Hankel functions in the expression for H_y^{feed} and G_{yy}^{WG} are always above unity ($kR \geq 2\pi$). We thus evaluate these Hankel functions with the following large-argument approximation ($\xi > 1$):

$$\mathrm{H}_{\eta}^{(2)}(\xi) \rightarrow \sqrt{\frac{2}{\pi\xi}} e^{-i\left(\xi - \frac{\eta\pi}{2} - \frac{\pi}{4}\right)}.$$

We initialize the bias variables ($b_p^{(1)}$ and $b_c^{(2)}$ in Figure 2 of the main text) as zero. Weight variables are initialized with a truncated normal distribution[1]; mean and standard deviation of the latter are zero and 0.12, respectively, for the digital weight variables ($w_{p,i}^{(1)}$ and $w_{c,p}^{(2)}$ in Figure 2 of the main text) and zero and 0.2, respectively, for the physical weight variables $t_{j,i}$.

To avoid issues related to division by zero, we use $\left\{t_j \alpha_{yy} + \delta\right\}^{-1}$ instead of $\left\{t_j \alpha_{yy}\right\}^{-1}$ as jth diagonal entry of $\mathbf{A}$, with δ being four orders of magnitude smaller than α_{yy}. A simple sanity check to confirm this procedure is presented in Figure S3. The magnetic dipole moments corresponding to a binary (random) metasurface configuration are computed and the result is compared to that obtained if elements in the "off" state are simply omitted. Ideally, the same magnetic dipole moments are computed for the "on" elements in both scenarios, which is indeed the case, as shown in Figure S3.

[1] The generated values follow a normal distribution with specified mean and standard deviation, except that values whose magnitude is more than 2 standard deviations from the mean are dropped and re-picked.

Training Algorithm and Parameters. – We use the MNIST dataset of handwritten digits[17] which consists of 60,000 samples of 28×28 gray-scale images of handwritten digits from 0 to 9 and 10,000 further such samples to test the learned network. For training, we split the training dataset and use 15% for validation purposes. Using a batch size of $n_{batch} = 100$ and the Adam method for stochastic optimization[18] with a step size of 10^{-3}, we train the weight variables of the network based on the training dataset, using the cross entropy between the known and predicted labels as error metric to be minimized. Every 50 iterations we compute the accuracy achieved on the validation dataset. We define a patience parameter to avoid overlearning on the training dataset: if the validation accuracy has not improved for seven consecutive times, we stop training. Finally, we evaluate the achieved accuracy on the completely unseen test dataset. This is the accuracy we report in the main text.

Since our measurements are complex-valued, we stack real and imaginary parts of the M measurements in a $1 \times 2M$ real-valued vector $\mathcal{M}$; then we feed $\mathcal{M}$ into the first fully connected layer. A subtle but crucial detail is the need to normalize $\mathcal{M}$. If its standard deviation is several orders of magnitude above or below unity, training the weights of the fully connected layers may take much longer or be unfeasible, in particular in combination with the temperature parameter (see below). Hence, we first identify a normalization constant as the average of the standard deviation of $\mathcal{M}$ obtained for ten different random TX and RX masks (obeying the chosen on/off constraint) using the training dataset. We then always divide $\mathcal{M}$ by that fixed normalization constant in the subsequent training, validation and testing. In other words, the normalization constant is fixed once and for all based on the training dataset.

The employed activation functions are defined for real-valued inputs as follows. A "rectified linear unit" (ReLu) activation function returns 0 if its argument x is negative, and otherwise the argument is returned unaltered:

$$f_{ReLu}(x) = \max(x, 0).$$

A SoftMax activation normalizes a p-element vector $\mathbf{Y} = [Y_1, Y_2, \dots, Y_p]$ into a probability distribution consisting of p probabilities whose sum yields unity:

$$f_{SoftMax}(Y_p) = \frac{e^{Y_p}}{\sum_p e^{Y_p}}.$$

Imposing a Discrete Distribution of Weights. – In this section, we provide the technical details of how we restrict the physical layer weights to be chosen from a list S of s predefined discrete values. For instance, the reconfiguration mechanism of the metamaterial elements

may leverage PIN-diodes such that a given element is either resonant or not at the working frequency f_0, which corresponds to a binary on/off amplitude modulation: $\mathcal{S}$ ={0,1}. Our procedure can also be directly applied to gray-scale tuning; for instance, in the case of a phased-array with 2-bit phase modulation we would use $\mathcal{S}$ ={1,i,-1,-i}. (Preliminary tests showed little gains in the sensing performance with gray-scale tuning compared to amplitude-binary tuning.)

The ANN weight variables are, however, trained by back-propagating errors[19] which relies on computing gradients. This approach is thus, at first sight, incompatible with variables that can only take a few discrete values. An elegant solution to this apparent problem consists in using a temperature parameter[20]. The general idea is to start with a continuously-distributed weight variable and to gradually force its distribution to become more and more discrete over the course of the iterations. By the end of the training, the weights are then effectively discrete and chosen from $\mathcal{S}$.

We define a scale factor β that increases according to a quadratic schedule with the number of iterations T:

$$\beta(T) = 1 + (\gamma T)^2,$$

where we use $\gamma = 0.0005$. For every variable t_i to be trained, we introduce an s-element vector $\mathbf{V_i} = [t_{i,1}, t_{i,2}, \dots, t_{i,s}]$. We then multiply the absolute value of $\mathbf{V_i}$ with the scale factor β and apply a SoftMax operation: $\mathbf{V'_i} = f_{SoftMax}(\beta|\mathbf{V_i}|)$. We go on to define t_i as a sum of its possible values $S_j \in \mathcal{S}$ weighted by $t'_{i,j}$ (the entries of $\mathbf{V'_i}$): $t_i = \sum_j t'_{i,j} S_j$. The entries of $\mathbf{V_i}$ are declared as weight variables and are trained via error back-propagation. The SoftMax function in combination with the gradually increasing scale factor ensures that eventually $\mathbf{V'_i}$ ends up having one unity entry and the remaining entries become zero. We thus gradually transition from a continuous to a discrete distribution. To ensure that the t_i variables are indeed binary after training, we defer checking the patience parameter until $\min\left(t'_{i,j}, 1 - t'_{i,j}\right)_{i,j}$ is below 0.0001.

The training convergence for a sample realization with *N*=64 and *M*=5 is shown in Figure S4. We display the accuracies evaluated in intervals of 50 iterations on the training batch to be used next (blue) and the validation dataset (red). Recall that the accuracy reported in the main text is not plotted in this figure, since it is evaluated on a test dataset that was in no way used during training. The training is stopped after 69 epochs in the example in Figure S4. While the validation accuracy saturates after roughly 20 epochs, we continue the training for much longer since at this stage the physical weights are not binary yet. Over the course of

the remaining iterations, the physical weights become more and more binary, and consequently physical and digital weights are continuously re-adjusted to ensure the high validation accuracy is maintained. A striking detail is the apparent periodicity of a dip in the blue training accuracy curve. This is easily understood: the training batches (100 samples per batch) cycle through the training data (51,000 samples), such that one batch with data that is somewhat harder to classify is re-seen after 510 iterations. Since we probe the accuracy only in intervals of 50 iterations, it will take 50/(510 mod 50) = 5 epochs until the same 'hard' batch is featured again on our plot.

Constrained Beam Synthesis / Wavefront Shaping. – This section is motivated by the wish to compare the performance of our Learned Integrated Sensing Pipeline not only with random illuminations but also with orthogonal and PCA-based illuminations. For PCA-based illuminations, M inverse design problems of beam synthesis have to be solved to identify metasurface configurations whose scene illuminations approximate as closely as possible the first M PCA modes. For orthogonal scene illuminations, a set of M configurations has to be identified such that their scene illuminations overlap as little as possible. Note that we do not impose a specific orthogonal basis (such as the Hadamard basis); the obtained illuminations with minimal mutual overlap will thus still look speckle-like. Both of these inverse design problems are notoriously difficult due to (i) the binary constraint and (ii) the inter-element coupling. Traditional approaches to tackle such NP-hard combinatorial optimization problems (even without the coupling constraint) include Gerchberg-Saxton algorithms[21] and semidefinite programming tools.

Here, we choose a much simpler approach resembling "adjoint"-based inverse-design methods[22,23]. We take the analog part of our pipeline in Figure 2 of the main text and define a new cost-function based upon $I_{z,i}(\boldsymbol{\zeta}) = E_{z,i}^{TX}(\boldsymbol{\zeta}) \cdot E_{z,i}^{RX}(\boldsymbol{\zeta})$ (since z is the dominant component). More specifically, we either maximize for a single mask the overlap with a principal scene component or minimize for a series of masks the average mutual overlap of the scene illuminations.

In order to identify M illuminations with minimal mutual overlap, we first compute the $M \times M$ overlap matrix $\boldsymbol{\Omega}$ whose (i,j)th entry is $\Omega_{i,j} = O\big(I_{z,i}(\boldsymbol{\zeta}), I_{z,j}(\boldsymbol{\zeta})\big)$, where O is the overlap function as defined in Equation S1 of the main text. Next, we compute the average mutual overlap as the average of the off-diagonal entries of $\boldsymbol{\Omega}$ and use that as cost function to be minimized: $\langle \Omega_{i,j} \rangle_{i \neq j}$.

In order to identify an illumination pattern that matches a given PCA mode $\mathcal{P}$ as closely as possible, we compute the overlap of the scene illumination I_z with $\mathcal{P}$ and use its inverse as cost function to be minimized: $1/O(I_z,\mathcal{P})$. Minimizing this cost function will maximize the resemblance of I_z to $\mathcal{P}$.

Using the temperature parameter as before ensures that during training the weights are carefully driven towards a binary distribution. This simple approach to deal with constraints and even coupling effects, only requiring the formulation of an analytical forward model, may also prove useful in other *constrained* physical inverse design problems, from nano-photonic inverse design[24] and optical wavefront shaping with digital micromirror devices[25] via beam synthesis with phased-arrays in the microwave domain[26,27] to infrared metamaterial phase holograms[28]. Note that we have an analytical forward model and borrow efficient error back-propagation tools (developed for neural network training) to solve a *constrained* inverse problem – in contrast to other recent efforts to solve *continuous* inverse problems by first training an ANN to approximate a forward model[8,9].

In passing, we thus introduce a simple constrained inverse configuration-design paradigm for dynamic metasurface apertures and show how it enables beam synthesis or the radiation of orthogonal patterns, as opposed to the (thus far) conventionally use of random patterns[29,30]. While the hardware is the same, the identification of appropriate metasurface configurations is an additional one-off effort. With a modified cost-function, one can also identify settings for scene illuminations with custom-tailored speckle statistics, which may drastically improve the efficiency of computational microwave ghost imaging[31–33].

III. Further Analysis of the LISP

A natural question that arises (albeit irrelevant for practical applications) is whether the other benchmark illumination schemes (random, orthogonal, PCA-based) will eventually, using more measurements, be able to perform as well as our LISP. For the N=16 case, we thus evaluated the average classification accuracy also for M=N and M=2N. Only the LISP curve had saturated; the other benchmarks accuracies were still slightly improving between M=N and M=2N and were still somewhat below the LISP performance. Since the "scene illumination" $\mathbf{I_i}(\boldsymbol{\zeta}) = \mathbf{E_i^{TX}}(\boldsymbol{\zeta}) \cdot \mathbf{E_i^{RX}}(\boldsymbol{\zeta})$ depends on the configuration of two metasurfaces with N tunable elements each, we expect that all schemes perform equally well only once $M \geq N^2$.

We observe no significant difference in the performance across different metasurface realizations (i.e. different random locations of the metamaterial elements). The LISP scene illuminations overlap around 65% across different realizations with the same metasurface, indicating that the optimization space contains numerous almost equivalent local minima. Remarkably, we never seem to get stuck in inferior local minima.

Robustness outside nominal conditions. – We investigate how robust the sensing performance is outside the nominal conditions, i.e. over a given set of parameter variations. Here, we consider variations of the metamaterial elements' polarizability; due to fabrication tolerances of electronic components such as the PIN diodes, the experimental polarizability is expected to differ across metamaterial elements by a few percent from the value extracted via full-wave simulation based on the element's design[13]. With M=5 and N=64, we first train our LISP as before. Then, for each metamaterial element, we replace the true polarizability value α_{yy} by a different $\alpha'_{yy} = \alpha_{yy}(1+\varepsilon)$, where ε is white noise; real and imaginary parts of ε are identically distributed with zero mean and standard deviation δ, so the standard deviation of ε (the size of the cloud in the complex plane) is $\sqrt{2}\delta$.

In Figure S5 we display the dependence of the sensing performance of our LISP scheme as a function of the polarizability fluctuations around the expected value. Mean and standard deviation over 250 realizations of fluctuations are shown in purple. Up to 5% of fluctuations, the performance does not display any notable changes; around 10% a very slight degradation of the performance is observed. The benchmark for comparison here is the case of using random illuminations since these do not rely on carefully shaped wavefronts and are thus not affected by experimental polarizability values different from the expected ones. Compared to the performance with random illuminations, our scheme is still clearly superior even for unrealistically high fluctuations on the order of 15%. These results suggest that realistic

deviations from the expected polarizability values of the metamaterial elements, due to fabrication tolerances or other neglected effects, are by no means detrimental for our scheme.

Effect of element failure. – We also consider the case in which one or several metamaterial elements fail, that is $\alpha'_{yy} = 0$ for the failing elements to mimic the loss of tunability. Without any measures to counteract the failure, the performance drops on average as shown in Figure S6. The standard deviation, as evidenced by the shaded area, is relatively large and in some cases the classification accuracy may be almost unaltered. This is expected when the polarizability of the failing elements was close to zero anyway or when they were predominantly configured into the "off" state.

It is important to note that Figure S6 considers the worst-case scenario of element failure without any compensation. A first compensation could consist in characterizing the new perturbed scene illuminations to then retrain the digital weights accordingly. A more advanced compensation would require the identification of the failing elements (which is relatively easy in practice) to then retrain the entire LISP and update both analog and digital weights. In the latter case, almost no loss in performance is expected: essentially, the situation is then equivalent to using a metasurface with fewer metamaterial elements and Figure 4 in the main text has shown that reducing the number of elements from 64 to 16 affects the achievable performance only slightly.

Finally, note that this discussion is equally valid for any of the other benchmark schemes since failing elements would result in scene illuminations different from the assumed ones in these schemes, too.

Effect of measurement noise. – Finally, we explore the performance of our LISP in the presence of measurement noise. To that end, we add white noise to the measurement vector $\mathcal{M}$ before feeding it into the digital layers. This, again, constitutes a worst-case scenario in which noise was not anticipated during LISP training. Otherwise, in line with the discussion in Section V, one can train the LISP to be robust to a certain level of noise by adding noise to the training dataset. Figure S7 shows that a LISP trained for noise-free operation yields satisfactory results for SNR levels above roughly 20 dB. All illumination benchmarks are expected to experience a degradation in performance in the presence of noise.

IV. Alternative Reconfigurable Hardware

Dynamic metasurface apertures similar to the one we consider in Figure 1a of the main text have previously been used to generate random scene illuminations for computational imaging[29,30]. In our present work, we take full advantage of the ability to individually control each radiating element to purposefully shape the scene illuminations. The individual addressability of each metamaterial element distinguishes these dynamic metasurfaces from other designs in the literature that simultaneously reconfigure all elements to redirect a beam[34–36].

Our LISP could of course also be implemented with other wavefront shaping setups such as arrays of reconfigurable antennas[37], reflect-arrays illuminated by a separate feed (e.g. a horn antenna)[38–40] or leaky-wave antennas with individually controllable radiating elements[41,42]. Indeed, if our concept was transposed to the acoustic or optical regime, one would probably use an array of acoustic transceivers or a spatial light modulator[43], respectively. In the microwave domain, however, antenna arrays are costly and the use of reflect-arrays yields bulky setups. In contrast, dynamic metasurface hardware as the one in Figure 1a of the main text only requires a single feed since it benefits from its inherent analog multiplexing and is moreover compact, planar and can be fabricated using standard PCB processes.

V. Outlook

In spite of preliminary results suggesting that our scheme's performance is robust to parameter variations (see Section III in the Supporting Information), it is worth considering how one would deal with significant fabrication inaccuracies in experimental metasurfaces. Should one or several parameters of the metasurface design, such as polarizabilities or locations of the metamaterial elements, turn out to significantly vary due to fabrication issues, an additional calibration step could easily learn the actual parameter values of a given fabricated metasurface. The spirit of this procedure is similar to the way in which we achieved beam synthesis (see Section II in the Supporting Information) using only the analog part of our pipeline. The parameters to be determined are declared as weight variables to be learned during training and initialized with their expected values. Then, experimentally the radiated fields for a few different metasurface configurations are measured and a cost function is defined to minimize during training the difference between the measured and expected radiated fields. By the end of the optimization, a set of parameter values will have been learned that optimally matches the experiment.

Furthermore, for practical applications it may be of interest to achieve a certain robustness to fluctuations in the calibration parameters such as geometrical details of the experimental setup[44,45]. By including random realizations of major calibration parameters within the expected range of fluctuation during training, the network can learn to be invariant to these variations[44]. Ultimately, this enables the transfer of knowledge learned on synthesized data to real-life setups without additional training measurements. For a specific task such as hand gesture recognition, synthetic scenes can easily be generated with appropriate 3D modelling tools.

A conceptually exciting question for future research is how we can conceive a LISP that is capable of making on-the-fly decisions about the next optimal scene illumination, taking into account the available knowledge from previous measurements[46].

Interestingly, our LISP can also be interpreted in light of recent efforts to meet the exploding computational demands of ANNs with wave-based analog computing, which seeks to perform desired operations with waves as they interact with carefully tailored systems[47–53]. Our wave-based sensing scheme is essentially a hybrid analog-digital ANN in which the interaction of learned optimal wavefronts with the scene acts as a first processing layer. As we have shown, data acquisition fulfills two processing tasks in our LISP, being (i) trainable and (ii) highly compressive. In the future, the digital electronic structures used to implement the

fully connected processing layers may be replaced by wave-based analog devices, as proposed in Refs.[48,49,52].

References

[1] B. T. Draine, P. J. Flatau, *J. Opt. Soc. Am. A* **1994**, *11*, 1491.
[2] A. D. Scher, E. F. Kuester, *Metamaterials* **2009**, *3*, 44.
[3] P. T. Bowen, T. Driscoll, N. B. Kundtz, D. R. Smith, *New J. Phys.* **2012**, *14*, 033038.
[4] N. Landy, D. R. Smith, *J. Appl. Phys.* **2014**, *116*, 044906.
[5] M. Dupré, P. del Hougne, M. Fink, F. Lemoult, G. Lerosey, *Phys. Rev. Lett.* **2015**, *115*, 017701.
[6] P. del Hougne, F. Lemoult, M. Fink, G. Lerosey, *Phys. Rev. Lett.* **2016**, *117*, 134302.
[7] T. Sleasman, M. F. Imani, J. N. Gollub, D. R. Smith, *Phys. Rev. Applied* **2016**, *6*, 054019.
[8] J. Peurifoy, Y. Shen, L. Jing, Y. Yang, F. Cano-Renteria, B. G. DeLacy, J. D. Joannopoulos, M. Tegmark, M. Solja, *Sci. Adv.* **2018**, 8.
[9] D. Liu, Y. Tan, E. Khoram, Z. Yu, *ACS Photonics* **2018**, *5*, 1365.
[10] G. Lipworth, A. Rose, O. Yurduseven, V. R. Gowda, M. F. Imani, H. Odabasi, P. Trofatter, J. Gollub, D. R. Smith, *Appl. Opt.* **2015**, *54*, 9343.
[11] L. Pulido-Mancera, M. F. Imani, P. T. Bowen, N. Kundtz, D. R. Smith, *arXiv:1807.11592* **2018**.
[12] I. Yoo, M. F. Imani, L. Pulido-Mancera, T. Sleasman, D. R. Smith, *IEEE Trans. Antennas Propag.* **2019**, *67*, 5853.
[13] L. Pulido-Mancera, P. T. Bowen, M. F. Imani, N. Kundtz, D. Smith, *Phys. Rev. B* **2017**, *96*, 235402.
[14] J. D. Jackson, *Classical Electrodynamics*, John Wiley & Sons, **1975**.
[15] G. W. Mulholland, C. F. Bohren, K. A. Fuller, *Langmuir* **1994**, *10*, 2533.
[16] M. Abadi, P. Barham, J. Chen, Z. Chen, A. Davis, J. Dean, M. Devin, S. Ghemawat, G. Irving, M. Isard, and others, *12th USENIX Symposium on Operating Systems Design and Implementation (OSDI 16)* **2016**, 265.
[17] Y. LeCun, L. Bottou, Y. Bengio, P. Haffner, *Proc. IEEE* **1998**, *86*, 2278.
[18] D. P. Kingma, J. L. Ba, *arXiv:1412.6980* **2014**.
[19] D. E. Rumelhart, G. E. Hintont, R. J. Williams, *Nature* **1986**, *323*, 533.
[20] A. Chakrabarti, *Advances in Neural Information Processing Systems* **2016**, 3081.
[21] J. R. Fienup, *Appl. Opt.* **1982**, *21*, 2758.
[22] J. S. Jensen, O. Sigmund, *Laser & Photon. Rev.* **2011**, *5*, 308.
[23] H. Chung, O. D. Miller, *arXiv:1905.09213* **2019**.
[24] A. Y. Piggott, J. Lu, K. G. Lagoudakis, J. Petykiewicz, T. M. Babinec, J. Vučković, *Nat. Photon.* **2015**, *9*, 374.
[25] A. Drémeau, A. Liutkus, D. Martina, O. Katz, C. Schülke, F. Krzakala, S. Gigan, L. Daudet, *Opt. Express* **2015**, *23*, 11898.
[26] H. Lebret, S. Boyd, *IEEE Trans. Signal Process.* **1997**, *45*, 526.
[27] B. Fuchs, *IEEE Antennas Wirel. Propag. Lett.* **2017**, *16*, 868.
[28] S. Larouche, Y.-J. Tsai, T. Tyler, N. M. Jokerst, D. R. Smith, *Nat. Mater.* **2012**, *11*, 450.
[29] T. Sleasman, M. F. Imani, J. N. Gollub, D. R. Smith, *Appl. Phys. Lett.* **2015**, *107*, 204104.
[30] M. F. Imani, T. Sleasman, D. R. Smith, *IEEE Antennas Wirel. Propag. Lett.* **2018**, *17*, 2299.
[31] N. Bender, H. Yılmaz, Y. Bromberg, H. Cao, *Optica* **2018**, *5*, 595.
[32] A. V. Diebold, M. F. Imani, T. Sleasman, D. R. Smith, *Optica* **2018**, *5*, 1529.
[33] K. Kuplicki, K. W. C. Chan, *Opt. Express* **2016**, *24*, 26766.
[34] R. Guzman-Quiros, J. L. Gomez-Tornero, A. R. Weily, Y. J. Guo, *IEEE Antennas Wirel. Propag. Lett.* **2012**, *11*, 1426.

[35] J. Fu, A. Li, W. Chen, B. Lv, Z. Wang, P. Li, Q. Wu, *IEEE Antennas Wirel. Propag. Lett.* **2017**, *16*, 760.
[36] K. Chen, Y. H. Zhang, S. Y. He, H. T. Chen, G. Q. Zhu, *IEEE Antennas Wirel. Propag. Lett.* **2019**, *18*, 551.
[37] A. J. Fenn, D. H. Temme, W. P. Delaney, W. E. Courtney, *Lincoln Lab. J.* **2000**, *12*, 20.
[38] J. Huang, J. A. Encinar, *Reflectarray Antennas*, John Wiley & Sons, **2008**.
[39] D. Sievenpiper, J. Schaffner, R. Loo, G. Tangonan, S. Ontiveros, R. Harold, *IEEE Trans. Antennas Propagat.* **2002**, *50*, 384.
[40] T. J. Cui, M. Q. Qi, X. Wan, J. Zhao, Q. Cheng, *Light Sci. Appl.* **2014**, *3*, e218.
[41] S. Lim, C. Caloz, T. Itoh, *IEEE Trans. Microwave Theory Techn.* **2004**, *52*, 2678.
[42] D. J. Gregoire, J. S. Colburn, A. M. Patel, R. Quarfoth, D. Sievenpiper, in *2014 International Conference on Electromagnetics in Advanced Applications (ICEAA)*, IEEE, Palm Beach, Aruba, **2014**, pp. 477–479.
[43] S. Rotter, S. Gigan, *Rev. Mod. Phys.* **2017**, *89*, 015005.
[44] G. Satat, M. Tancik, O. Gupta, B. Heshmat, R. Raskar, *Opt. Express* **2017**, *25*, 17466.
[45] A. Mutapcic, S. Boyd, A. Farjadpour, S. G. Johnson, Y. Avniel, *Eng. Optimiz.* **2009**, *41*, 365.
[46] V. Mnih, N. Heess, A. Graves, K. Kavukcuoglu, *Advances in Neural Information Processing Systems* **2014**, 2204.
[47] A. Silva, F. Monticone, G. Castaldi, V. Galdi, A. Alu, N. Engheta, *Science* **2014**, *343*, 160.
[48] Y. Shen, N. C. Harris, S. Skirlo, M. Prabhu, T. Baehr-Jones, M. Hochberg, X. Sun, S. Zhao, H. Larochelle, D. Englund, M. Soljačić, *Nat. Photon.* **2017**, *11*, 441.
[49] P. del Hougne, G. Lerosey, *Phys. Rev. X* **2018**, *8*, 041037.
[50] N. Mohammadi Estakhri, B. Edwards, N. Engheta, *Science* **2019**, *363*, 1333.
[51] F. Zangeneh-Nejad, R. Fleury, *Nat. Commun.* **2019**, *10*, 2058.
[52] T. W. Hughes, I. A. D. Williamson, M. Minkov, S. Fan, *arXiv:1904.12831* **2019**.
[53] D. Pierangeli, G. Marcucci, C. Conti, *Phys. Rev. Lett.* **2019**, *122*, 213902.

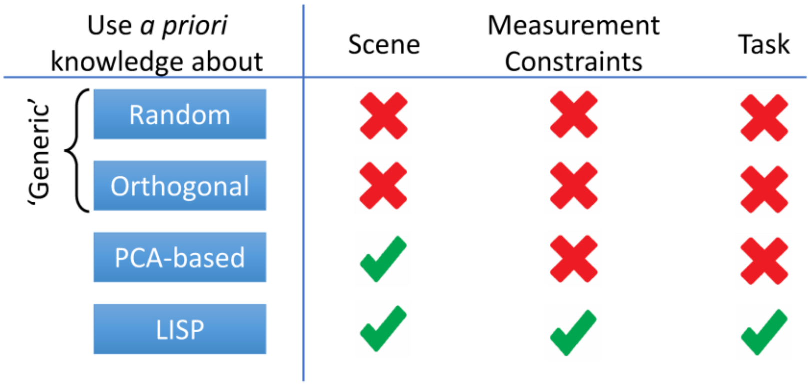

Figure S1. Taxonomy of illumination strategies in wave-based sensing in terms of the *a priori* knowledge they make use of. Schemes using generic (random or orthogonal) scene illuminations ignore any available *a priori* knowledge. Illuminations based on a principle component analysis (PCA) of the scene include knowledge about the scene but not about the hardware constraints or task to be performed. Our proposed Learned Integrated Sensing Pipeline (LISP) makes use of all available knowledge by integrating measurement and processing into a unique ML pipeline.

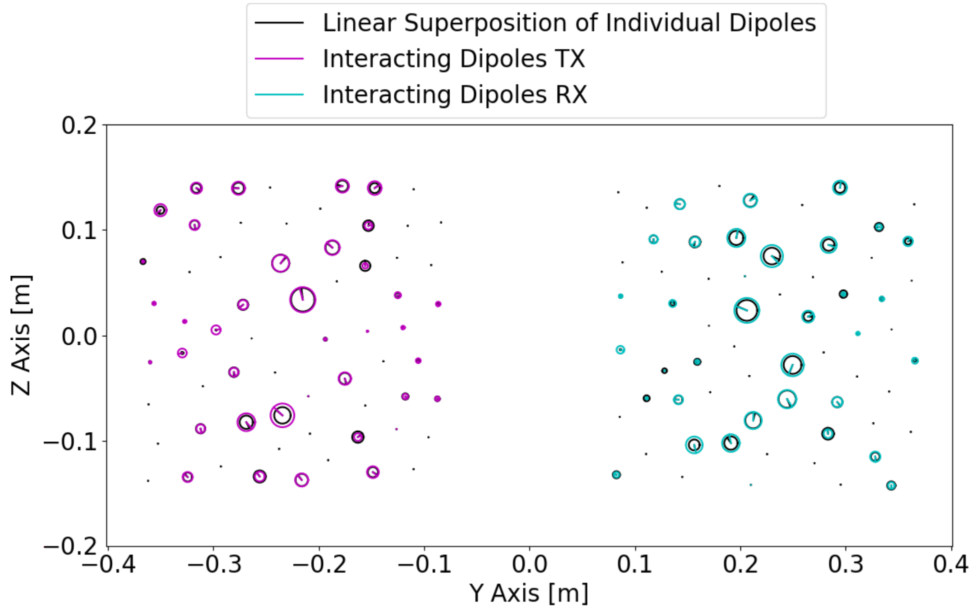

Figure S2. Visualization of coupling between metamaterial elements. We compare the dipole moments m_y computed for each metamaterial element, without (black) and with (purple/cyan) accounting for the coupling between elements. The dipole moments are represented as phasors, with the radius of the circle being proportional to their amplitude, and the line segment showing their phase. The circles are centered on the physical location of each metamaterial element.

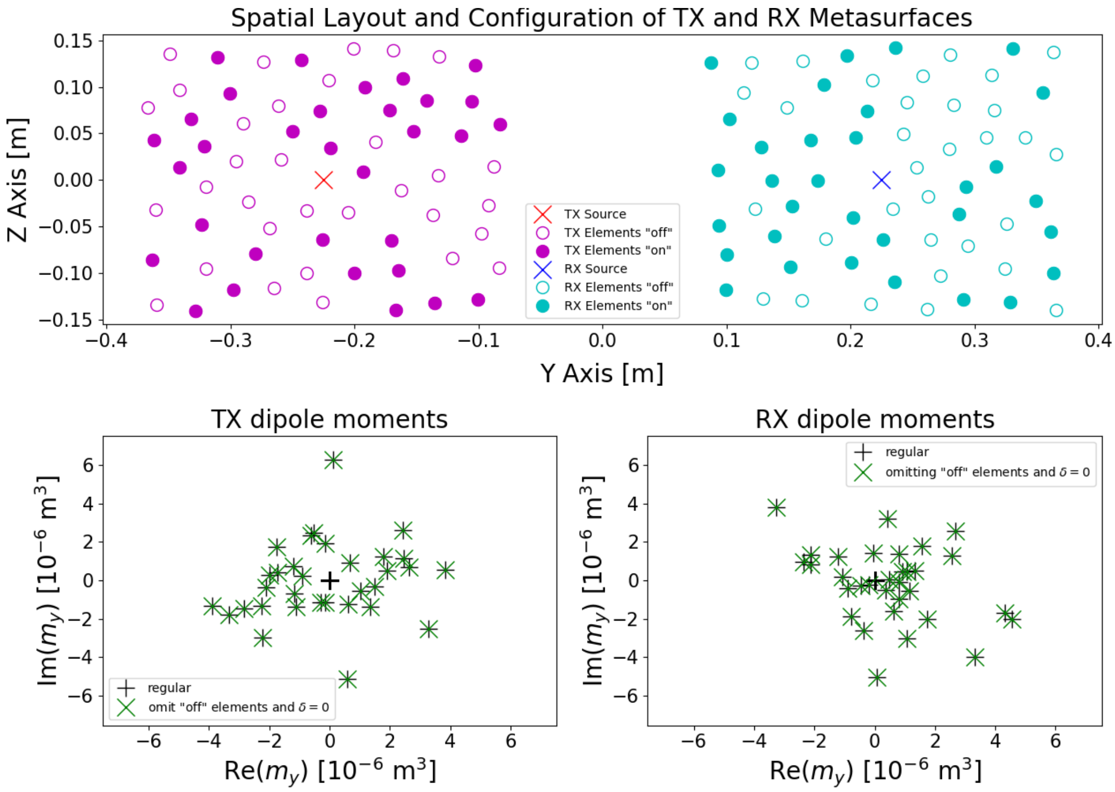

Figure S3. Sanity check of the tuning mechanism. The top figure shows the spatial layout of the considered 64-element metasurfaces, as well as the chosen (random) configurations: filled elements are "on", unfilled elements are "off". The bottom row contrasts the computed dipole moments for the two metasurfaces, once using $\{t_j \alpha_{yy} + \delta\}^{-1}$ as entries of the diagonal of **A** (black) and once omitting the "off" elements and using $\{\alpha_{yy}\}^{-1}$ as entries of the diagonal of **A** (green). Excellent agreement is seen.

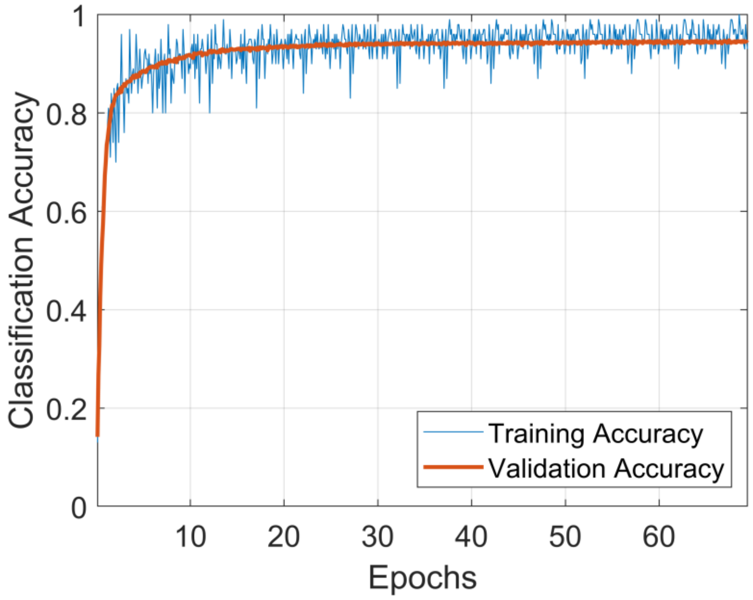

Figure S4. Training convergence for a sample realization with *N*=64 and *M*=5. The blue curve indicates the accuracy achieved on the training batch to be used next. The red curve indicates the accuracy achieved on the validation dataset. The curves sample the optimization dynamics in steps of 50 iterations.

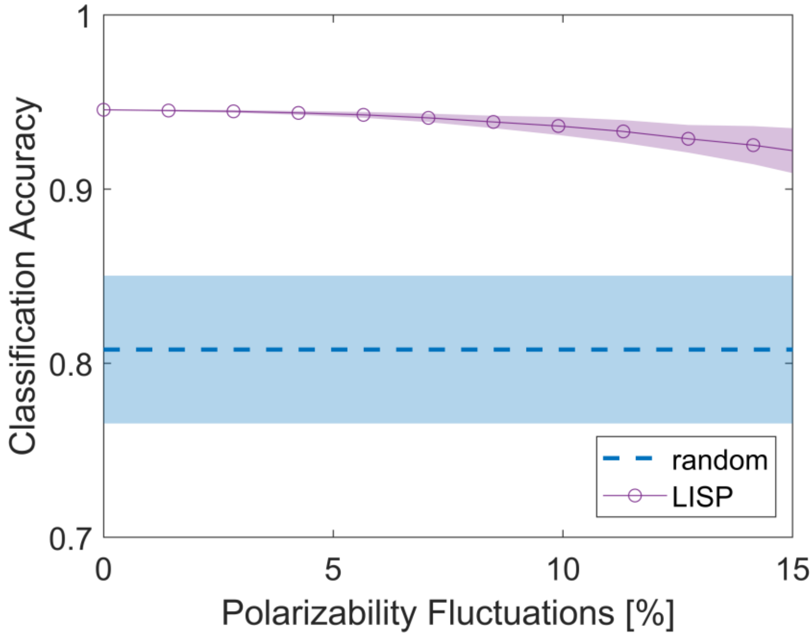

Figure S5. Robustness of sensing performance to fluctuations in the metamaterial elements' experimental polarizability. For a single realization with N=64 and M=5, the LISP is trained with the expected polarizability value. Then, its performance is evaluated after adding different amounts of white noise to the metamaterial elements' polarizabilities. The purple curve and shaded area indicate average and standard deviation over 250 realizations of the polarizability fluctuations. For reference, the blue curve and shaded area indicate the average performance with random metasurface configurations, see Figure 4a of the main text, which is independent of polarizability fluctuations since it does not rely on optimized wavefronts.

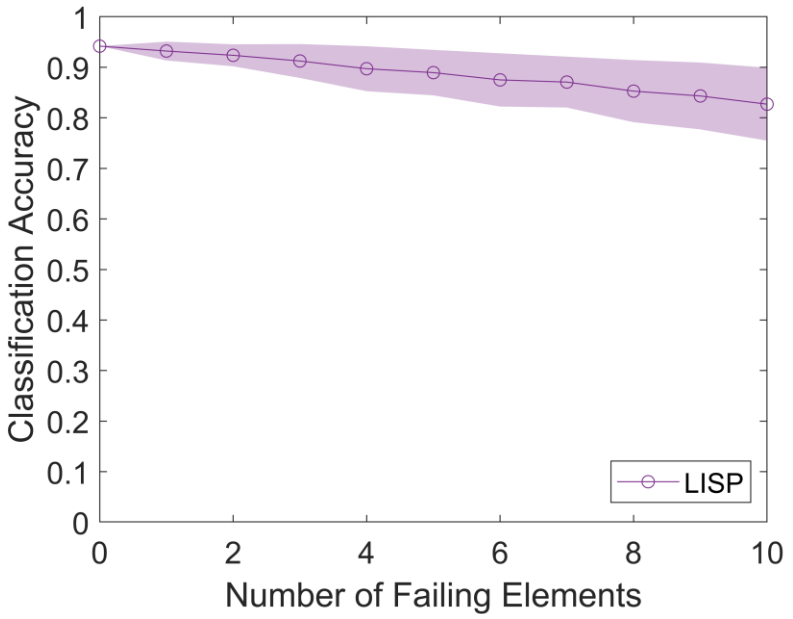

Figure S6. Worst-case scenario of element failure without any compensation. The LISP is trained with N=64 and M=5. Then, its performance is evaluated after setting the polarizability of one or several randomly selected metamaterial elements to zero, mimicking the loss of tunability due to element failure. The purple curve and shaded area indicate, respectively, average and standard deviation over 500 realizations of random selections of the failing element(s). As discussed in Section III in the Supporting Information, this is the worst-case scenario: by retraining the entire LISP the performance can be expected to remain virtually unaltered.

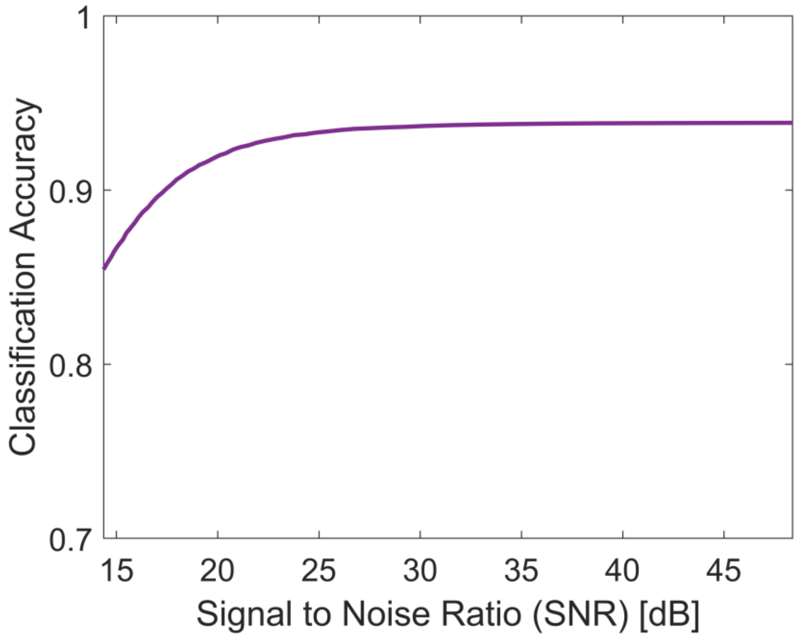

Figure S7. Impact of noise on the performance of a LISP trained for noise-free operation. The LISP is trained for noise-free operation with N=64 and M=5. Then, its performance is evaluated after adding different amounts of white noise to the measurements obtained with the unseen test dataset. The signal-to-noise ratio (SNR) of the measurements is indicated on the horizontal axis. The purple curve shows the average over 100 realizations of the measurement noise, the standard deviation is negligible and hence not visible.
